# 5-FU-Induced Upregulation of Exosomal PD-L1 Causes Immunosuppression in Advanced Gastric Cancer Patients

**DOI:** 10.3389/fonc.2020.00492

**Published:** 2020-04-22

**Authors:** Min Zhang, Yibo Fan, Xiaofang Che, Kezuo Hou, Chaoxu Zhang, Ce Li, Ti Wen, Shuo Wang, Yu Cheng, Yunpeng Liu, Xiujuan Qu

**Affiliations:** ^1^Department of Medical Oncology, The First Affiliated Hospital of China Medical University, Shenyang, China; ^2^Key Laboratory of Anticancer Drugs and Biotherapy of Liaoning Province, The First Affiliated Hospital of China Medical University, Shenyang, China

**Keywords:** exosomal PD-L1, 5-fluorouracil, gastric cancer, immunosuppression, nivolumab

## Abstract

Although the cytotoxic chemotherapeutic agent 5-fluorouracil (5-FU) is generally considered to directly kill cancer cells and exert immunostimulatory effects in advanced gastric cancer, accumulating evidence indicates that it upregulates the expression of PD-L1, a representative immune checkpoint blockade molecule involved in negative regulation of the immune response. It was reported that exosomes could transfer functional PD-L1 locally and distantly to suppress the antitumor immune response. However, whether 5-FU alters the expression of exosomal PD-L1 and induces immunosuppression in gastric cancer remains unclear. Herein, we found that 5-FU increased gastric cancer-derived exosomal PD-L1. Importantly, compared with baseline levels, circulating exosomal PD-L1 was significantly upregulated in 21 stage III–IV gastric cancer patients after two, four, and six repeated cycles of fluoropyrimidine treatment (*P* = 0.009, *P* = 0.047, and *P* = 0.023, respectively), accompanied by decreased amounts of IFN-γ, TNF-α, IL-2, IL-6, and GM-CSF (*P* = 0.014, *P* = 0.004, *P* = 0.009, *P* = 0.031, and *P* = 0.014, respectively). Additionally, circulating exosomal PD-L1 was increased more significantly in clinical non-responders compared with responders (*P* = 0.018). Furthermore, exosomal PD-L1 induced apoptosis in Jurkat T cells and inhibited T cell activation in PBMCs, which could be partly reversed by nivolumab. These results suggested that 5-FU-induced upregulation of exosomal PD-L1 causes systemic immunosuppression in advanced gastric cancer following multiple cycles of chemotherapy, especially after two cycles.

## Introduction

Gastric cancer (GC) is the fifth most prevalent cancer and the third leading cause of cancer-related death worldwide. In 2018, GC caused 782,685 deaths (1). The majority of GC patients show advanced to late-stage disease at the time of diagnosis because efficient early diagnostic markers are unavailable (2). Chemotherapy, one of the main treatment methods, has been extensively applied in advanced gastric cancer. Accumulating evidence indicates that, although conventional chemotherapeutic drugs exert direct cytostatic/cytotoxic effects (3), they could alter tumor-reactive immune responses and even display immunosuppressive features (4), which may affect the survival of patients. 5-FU is the backbone substance of advanced gastric cancer chemotherapy (5). 5-FU kills tumor cells through misincorporation of fluoronucleotides into DNA and RNA molecules and by inhibition of the nucleotide synthesis enzyme thymidylate synthase (TS) (6). 5-FU was shown to selectively eliminate tumor-associated MDSCs resulting in enhanced T-cell-dependent antitumor immunity in several types of cancer (7). Nevertheless, a better understanding of the mechanism by which 5-FU may alter antitumor immune responses in gastric cancer patients, especially immune status changes after different cycles of 5-FU treatment, is important for the improvement of treatment efficacy.

Substantial evidence suggests that some chemotherapeutic drugs exert immunogenic effects that could improve the antitumor response (3, 8) by the following: (1) promoting antigen presentation and recognition; (2) provoking immunogenic cell death (ICD) (9, 10); (3) increasing the amount of effector T cells; (4) reducing immunosuppressive cells, including MDSCs, regulatory T cells, and type II macrophages; and (5) regulating the secretion of cytokines. In recent years, the immunosuppressive effects of some chemotherapeutic drugs have been noticed, while much attention has been paid to the immunostimulatory aspect of chemotherapy (10). Several studies have suggested that chemotherapeutic drugs could impair antitumor immunity mainly in the following aspects: (1) reducing circulating immune cells, (2) inducing immunosuppressive monocytic myeloid cells (10), (3) upregulating PD-L1 to induce T cells anergy and immune evasion by interacting with PD-1 on activated T cells (11, 12), and (4) triggering chemotherapy-elicited extracellular vesicles (EVs) to exert prometastatic effects (13). Interestingly, it was reported that the doses and times of chemotherapeutic drugs as well as the methods of administration *in vivo* or *in vitro* might alter the immune system differently (14). Two studies have shown that 5-FU increases PD-L1 levels in breast and colorectal cancer cells, but little is known about PD-L1 level changes associated with 5-FU in gastric cancer. Moreover, whether 5-FU concentration and treatment time affect PD-L1 in tumor cells and the immune system in advanced gastric cancer remains largely unknown.

Exosomes represent membrane-bound organelles containing various bioactive molecules and play a key role in intercellular communication, affecting physiological functions; in addition, they can “package” DNA, RNA, and proteins of tumor cells to the local or systemic body, facilitating tumor progression and metastasis (15–17). Besides membrane-bound and extracellular soluble forms, several studies have demonstrated that PD-L1 also has exosomal forms (18–20). In addition, Mauro et al. confirmed that tumor-derived exosomes presenting PD-L1 could migrate to the draining lymph node, inducing local and/or systemic immunosuppression that promotes tumor progression (20). Our previous study revealed that exosomal PD-L1 is stable and might have stronger immunosuppressive activity than other forms of PD-L1 (21). Thus, we hypothesized that 5-FU may not only change PD-L1 expression in gastric cancer but also alter tumor-derived exosomal PD-L1, exerting stronger and more extensive immunosuppressive effects.

In the present study, we investigated the effect of 5-FU on exosomal PD-L1 in patients with advanced gastric cancer. Clinical blood samples at baseline and after different cycles of treatment with fluoropyrimidine were used to assess the effects of fluoropyrimidine on circulating exosomal PD-L1 and the immune status. Compared with baseline levels, circulating exosomal PD-L1 was upregulated after two, four, and six cycles of fluoropyrimidine treatment (*P* = 0.009, *P* = 0.047, and *P* = 0.023, respectively). Cytokines including interferon-γ (IFN-γ), tumor necrosis factor-α (TNF-α), interleukin (IL)-2, IL-6, and granulocyte-macrophage colony stimulating factor (GM-CSF) were decreased obviously following repeated cycles chemotherapy, especially after two cycles (*P* = 0.014, *P* = 0.004, *P* = 0.009, *P* = 0.031, and *P* = 0.014, respectively). Further exploration demonstrated that tumor-derived exosomal PD-L1 induced apoptosis in Jurkat T cells and inhibited the activation of T cells in peripheral blood mononuclear cells (PBMCs) *in vitro*.

## Materials and Methods

### Patients and Plasma Collection

This retrospective study was conducted on stage III–IV gastric cancer patients (*N* = 21), who were followed up at the First Hospital of China Medical University from 2013 to 2018. Patients were enrolled into the cohort if they met the following criterion: only received fluoropyrimidine standardized monochemotherapy (including 5-FU, Capecitabine, S-1). The study was approved by the Ethics Committee of China Medical University, and all research were conducted in accordance with ethical principles. The clinical characteristics and details of all patients were retrieved from the Hospital Information System. Samples of peripheral blood were collected from the patients into separator tubes immediately before and after chemotherapy, which were subjected to centrifugation at 3,000 rpm for 20 min at 4°C to isolate plasma for preparing plasma-derived exosomes.

### Cell Culture

Human gastric cell lines MGC803, SGC7901, and AGS were obtained from the Type Culture Collection of the Chinese Academy of Sciences (Shanghai, China). MKN74 was obtained from JCRB (Osaka, Japan). MKN45 and NCI-N87 cells were obtained from ATCC (Maryland, USA). Jurkat T cells were obtained from the American Type Culture Collection (ATCC, Rockville, MD, USA). All cells were maintained in Roswell Park Memorial Institute (RPMI)-1640 medium (Gibco, Gaithersburg, MD, USA) containing 10% heat-inactivated fetal bovine serum (FBS), penicillin (100 U ml^−1^), and streptomycin (100 mg ml^−1^) in a humidified incubator with a mixture of 95% air and 5% CO_2_ at 37°C. Cells were passaged every 2–3 days when they were 80% confluent and were tested monthly for mycoplasma contamination.

### Exosomes Purification

Exosomes were isolated from plasma samples of gastric cancer patients using Exosome Precipitation Solution (ExoQuick™, SBI) following the manufacturer's instructions. Exosomes from supernatants of gastric cells were isolated by ultracentrifugation as described previously (22). MKN74 cells were cultured in RPMI-1640 medium with exosome-deleted FBS (depleted of bovine exosomes by overnight centrifugation at 100,000 × g at 4°C). We collected cultured conditioned medium after 48 h and centrifuged at 3,000 × g for 15 min to remove cellular debris and dead cells. Then, the supernatant was filtered through 0.22 μM pore filter to eliminate larger extracellular vesicles. Exosomes were pelleted by ultracentrifugation at 100,000 × g for 70 min for differential centrifugation. The exosomes were washed with phosphate-buffered saline (PBS) and were centrifugated at 100,000 × g for 70 min once again, then resuspended in PBS and stored at −80°C. Concentration of exosomes was determined by protein quantification (Micro BCA Protein Assay Kit; Thermo Scientific, Waltham, MA, USA) according to the manufacturer's instructions.

### Transmission Electron Microscopy

Exosomes derived from MKN74 were fixed in 4% paraformaldehyde and placed on a formvar carbon-containing grid, contrasted, and then embedded in a mixture of uranyl acetate and methylcellulose, followed by observation under a JEM-1200EX transmission electron microscope (JEOL, Tokyo, Japan). We captured images at 70,000 × amplification.

### NanoSight Measurements

Analysis of absolute size distribution of isolated exosomes was detected by a NanoSight NS300 instrument (Malvern Instruments, Malvern, UK) following the manufacturer's protocol.

### Enzyme-Linked Immunosorbent Assay

The expression of soluble PD-L1 in cultured cells and PD-L1 in plasma-derived exosomes were detected by enzyme-linked immunosorbent assay (ELISA) (DY156, R&D), as recommended by the manufacturer's protocol. The optical density (OD) value of each well was measured under 450 nm by Bio-RAD iMark Microplate Reader (Bio-RAD Laboratories Inc., Kyoto Japan). The concentration of PD-L1 was quantitated by standard curve.

### Cytokine Measurement Analysis

The expression of cytokine in the plasma of gastric cancer patients was profiled by multiplex bead assay because of the high sensitivity. The experiment was performed using Bio-Plex Pro Human Cytokine 8-plex Assay (M50000007A, Bio-Rad). The detected cytokines included IL-2, IL-4, IL-6, IL-8, IL-10, IFN-γ, GM-CSF, and TNF-α, the detection of which was conducted following the manufacturer's instruction. Bio-Plex suspension array system and Bio-Plex Manager software measured the concentration of cytokines.

### Development of PD-L1-Knockdown Cells

Lentiviral particles with short hairpin RNAs (shRNAs) targeting human PD-L1 gene (PD-L1-KD group) or scrambled shRNA control (NC group) cotransfected MKN74 cells with viral packing plasmid and PolyFect Transfection reagent (OBiO Technology, Shanghai, China) were performed according to the manufacturer's instruction. ShRNA sequences for PD-L1 were as follows: 5′-CCAGCACACUGAGAAUCAA-3′ (sense), 5′-UUGAUUCUCAGUGUGCUGG-3′ (antisense). MKN74 cells (2 × 10^3^/well) were cultured in a 96-well-plate overnight and then transfected at a multiplicity of infection (MOI) of 100, respectively. After transfection for 48 h, we used fluorescence microscopy to validate the infection efficiency >90%. PD-L1-KD cells were selected in the presence of 2 μg/ml puromycin (Sigma-Aldrich) for 48 h, validated by Western blot. We harvest and cultured the MKN74 PD-L1-KD cells for exosomes preparation.

### Small Interfering RNA Transfections

STAT5A and Cbl-b small interfering RNA (siRNA) and the negative control (NC) were obtained from GeneChem (Shanghai, China). The sequences were as follows: STAT5A, 5′-AUGGAUAUGUGAAACCACAttUGUGGUUUCACAUAUCCAUca-3′ and Cbl-b, 5′-CCTGATGGGAGGAGTTATA-3′; NC, 5′-UUCUCCGAACGUGUCACGUttACGUGACACGUUCGGAGAAtt-3′. MKN74 or MGC803 cells (1.5 × 10^5^/well) were respectively cultured in six-well-plates overnight and then were transfected with siRNA or NC using lipofectamine 2000 (Invitrogen, Carlsbad, CA, USA) according to the manufacturer's instructions, performed as previously described. After 48 h of incubation with the transfected mix, the gene-silencing efficiency was verified by Western blot.

### Western Blot

The protein from cells and exosomes were detected by Western blot, performed as previously described (23). The target bands of proteins were detected with an enhanced chemiluminescence reagent (SuperSignal Western Pico Chemiluminescent Substrate, Pierce; Thermo Fisher Scientific, Inc.) and were scanned by the Electrophoresis Gel Imaging Analysis System (DNR Bio-Imaging Systems, Neve Yamin, Israel) and analyzed by NIH Image J software. Antibodies against glyceraldehyde 3-phosphate dehydrogenase (GAPDH) (25778), STAT5A (1081), and Cbl-b (#sc8006) were purchased from Santa Cruz Biotechnology (Santa Cruz, CA, USA). Antibodies specific to PD-L1 (13684S), phospho-STAT4 (5267S), phospho-STAT5 (9351S), and STAT4 (2653) were from Cell Signaling Technology (Danvers, MA, USA). Antibodies against CD9 (ab92726) and CD63 (ab193349) were from Abcam (Danvers, MA, USA).

### RNA Isolation and Reverse Transcription-Quantitative Real-Time PCR

The isolation of total RNA was performed as previously described (23). For microRNAs, The One Step PrimeScript® miRNA cDNA Synthesis Kit (Takara, Japan) was used for RNA reverse transcription. The comparative cycle threshold (Ct) method was used to calculate relative expression of miR-940, and the expression of U6 small nuclear RNA was used as reference. The forward primer for miR-940 was 5′-AAGGTTTAGCCGCTCCCCAAA-3′ and that for U6 internal control was forward 5′-GCTTCGGCAGCACATATACTAAAAT-3′ and reverse 5′-CGCTTCACGAATTTGCGTGTCAT-3′, respectively. The Uni-miR qPCR Primer was included in the kit. SYBR® Premix Ex Taq™ II (Perfect Real Time) (Takara, Japan) was used for monitoring the amount of microRNA (miRNA). The PCR conditions were 30 s at 95°C, followed by 45 cycles at 95°C for 5 s and 58°C for 25 s. The threshold cycle and 2^−ΔΔ*Ct*^ method were used for calculating the relative amount of the target RNA.

### Flow Cytometry Analysis

PD-L1 on the membrane of MKN74 and MGC803 was detected by flow cytometry. Briefly, cells were incubated with PE antihuman CD274 (557924, BD) for membrane staining for 20 min at 4°C and were washed with PBS. Acquisition of 10,000 events in the gated population was performed in BD Accuri C6. The expression of CD9 on the membrane of MKN74-derived exosomes was detected by flow cytometry. Briefly, exosomes were incubated with aldehyde/sulfate latex beads (A37304, Thermo Fisher Scientific) in PBS and then added with 1 M glycine and 10% bovine serum albumin (BSA), respectively. Exosomes were incubated with PE antihuman CD9 (555372, BD) for membrane staining and were detected by BD Accuri C6. Isotype control Ab staining was used as a negative control.

### PBMCs Separation and Cell Culture

PBMCs were obtained from healthy donors (*N* = 3) and were isolated from venous blood (5 ml) by density gradient centrifugation (Allegra X-15R Centrifuge, Beckman Coulter). Briefly, blood diluted in PBS was layered over Ficoll–Paque (Sinopharm Chemical Reagent Co., Ltd, China) and centrifuged at 400 × g for 30 min at 25°C. The mononuclear cell band was recovered and washed twice in PBS. After separation, PBMCs were maintained in RPMI-1640 medium containing 10% heat-inactivated FBS, penicillin (100 U ml^−1^), and streptomycin (100 mg ml^−1^) in a humidified incubator with a mixture of 95% air and 5% CO_2_ at 37°C.

### Function Analysis

Aliquots (2 × 10^5^) of PBMCs were placed in wells of 96-well-plates, and T cells in PBMCs were activated by human CD3/CD28 T cell activator (#10971, Stemcell Technologies) for 6 h at 37°C and then coincubated with MKN74-derived NC exosomes or PD-L1-KD exosomes for 48 h in the presence or absence of nivolumab (Bristol-Myers Squibb Company, Princeton, NJ, USA). PBMCs were stained with the following antibodies: FITC antihuman CD3 (561806, BD), APC antihuman CD69 (560967, BD), and PE antihuman CD25 (560989, BD). Isotype control Ab staining was used as a negative control.

### Cell Apoptosis Assay

Jurkat T cells (3 × 10^5^/well) were activated with 50 ng/ml phorbol 12-myristate 13-acetate (PMA; Sigma-Aldrich) for 24 h and then were coincubated with MKN74-derived NC exosomes or PD-L1-KD exosomes (200 μg/ml) for 48 h. Then, cells were harvested and stained using an Annexin V-fluorescein isothiocyanate/propidium iodide apoptosis detection kit (BMS500FI-100; Invitrogen; Thermo Fisher Scientific, Inc.) in the dark at room temperature. After being washed and resuspended in PBS, the samples were determined and analyzed by BD Accuri C6.

### Bioinformatic Analysis

UCSC Genome Browser (http://www.genome.ucsc.edu/) predicted DNA sequences in promoter regions of PD-L1. Then, the Promoter 2.0 Prediction Server (http://www.cbs.dtu.dk/services/Promoter/) predicted transcription factors that interact with sequences in promoter regions within a dissimilarity margin ≤ 15%. Animal TFDB 3.0 (https://bioinfo.life.hust.edu.cn/Animal TFDB/#!/) also predicted transcription factors according to the predicted DNA sequences, and there were 15 transcription factors in common. Correlation of mRNA-seq data of predicted transcription factors and PD-L1 was evaluated based on the TCGA-STAD RNA-Seq-HTSeq-FPKM database from The Cancer Genome Atlas (TCGA, https://tcga-data.nci.nih.gov/docs/publications/tcga/) and the gene expression profile of GSE62254 in GC from the NCBI-GEO database (http://www.ncbi.nlm.nih.gov/geo/) by Pearson correlation coefficient, as appropriate.

### Data and Statistical Analysis

The experimental results were reported as mean ± standard deviation (SD), and the mean values were calculated from more than three independent experiments. SPSS 16.0 software (IBM, USA) was used for all statistical analyses. GraphPad-Prism version 6.0 (GraphPad Software, USA) was used to perform graphics. Comparisons between two groups were performed using the Student's *t*-test. Comparisons between multiple groups were performed using the one- or two-way ANOVA. *P* < 0.05 indicated that there existed statistical differences (^*^P < 0.05, ^**^P < 0.01, ^***^P < 0.001, ^****^P < 0.0001).

## Results

### 5-FU Upregulates PD-L1 in Human Gastric Cancer Cells

**Figure 1:**
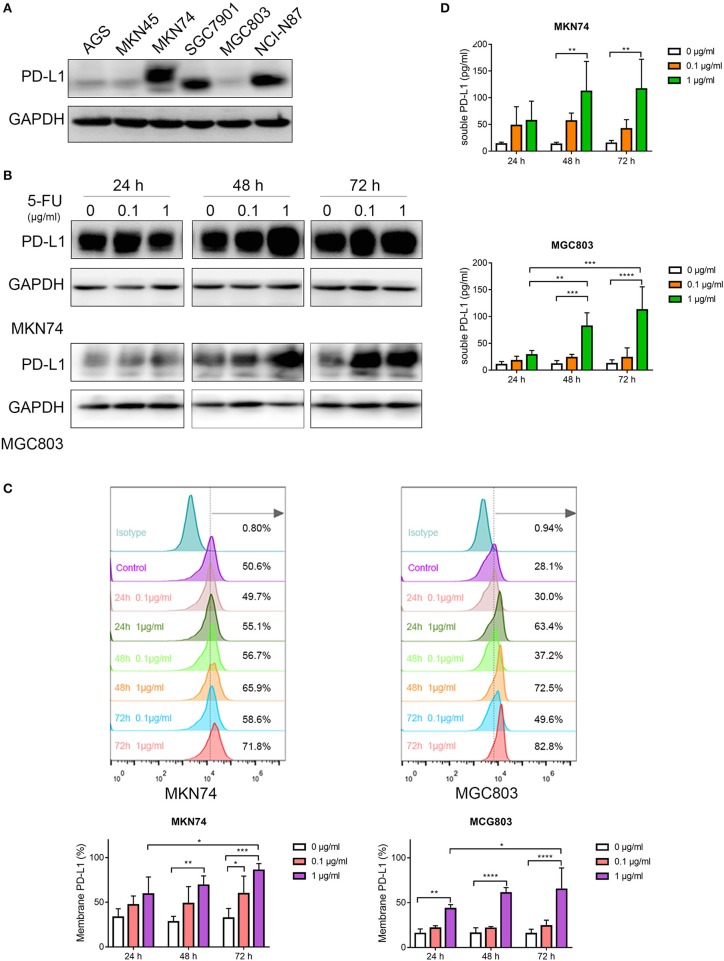
5-Fluorouracil (5-FU) upregulates programmed death-ligand 1 (PD-L1) in human gastric cancer cells. **(A)** Western blot was performed to measure PD-L1 levels in gastric cancer cell lines, including AGS, MKN45, MKN74, SGC7901, MGC803, and NCI-N87 cells. MKN74 and MGC803 cells were incubated with 5-FU (0, 0.1, 1.0 μg/ml) for 24, 48, and 72 h, respectively. **(B)** Western blot detected PD-L1 expression in MKN74 and MGC803 cells. **(C)** PD-L1 levels on the membrane of MKN74 and MGC803 cells were measured by flow cytometry. **(D)** Soluble PD-L1 levels in the cell culture supernatant were detected by ELISA. Data represent mean ± SD, *P* < 0.05 indicates that there existed statistical differences (*P < 0.05, **P < 0.01, ***P < 0.001, ****P < 0.0001). Data were representative of three independent experiments.

To analyze PD-L1 expression in gastric cancer cell lines, Western blot was performed to measure PD-L1 levels in AGS, MKN45, MKN74, SGC7901, MGC803 and NCI-N87 cells. As shown in Figure 1A, MKN74 cells expressed high levels of PD-L1, while low levels of PD-L1 were detected in MGC803 cells. Since previous studies have demonstrated that chemotherapy treatment might increase PD-L1 expression in some cancer types (11, 24, 25), we aimed to explore whether 5-FU has a similar effect in gastric cancer. The gastric cancer MKN74 and MGC803 cell lines, with high and low expression of PD-L1, respectively, were incubated with 5-FU (0, 0.1, 1.0 μg/ml), and then, PD-L1 level was detected at the 24, 48, and 72 h time points. Western blot showed that PD-L1 levels in both MKN74 and MGC803 cells were increased by 5-FU in a dose- and time-dependent manner, with maximum value obtained after treatment with 5-FU at 1.0 μg/ml for 72 h; meanwhile, 5-FU-induced PD-L1 upregulation was more pronounced in MGC803 cells compared with MKN74 cells (Figure 1B). Additionally, 5-FU increased membrane expression of PD-L1 in MKN74 and MGC803 cells, in a dose- and time-dependent manner, as detected by flow cytometry (Figure 1C). It was reported that besides membrane-bound forms, PD-L1 also has soluble forms (26–28). 5-FU also increased the amounts of soluble PD-L1 in cell culture supernatants, as detected by ELISA (Figure 1D). Taken together, these results showed that 5-FU upregulated PD-L1 in a dose- and time-dependent manner in gastric cancer cell lines.

### 5-FU Increases the Secretion of Exosomal PD-L1

**Figure 2:**
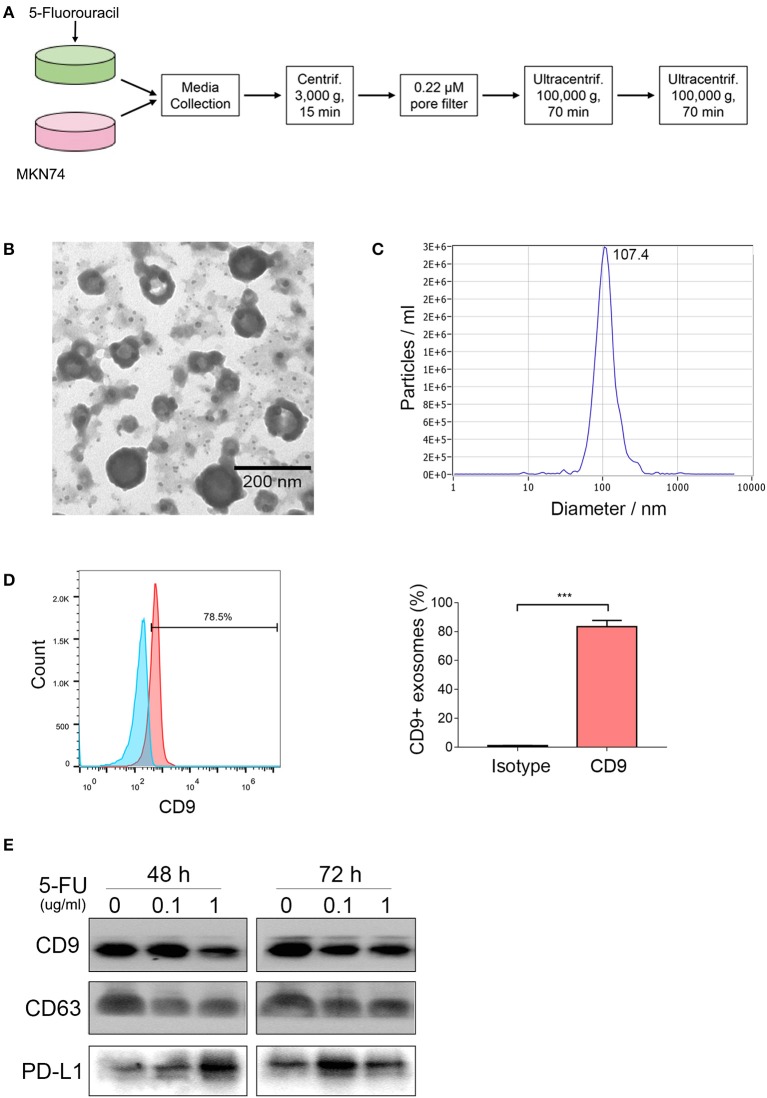
5-Fluorouracil (5-FU) increases the secretion of exosomal programmed death-ligand 1 (PD-L1). MKN74 cells were incubated with 5-FU (0, 0.1, 1.0 μg/ml) for 48 and 72 h, respectively. **(A)** Schematic illustration of MKN74 cell-derived exosomes isolation and purification from the supernatants by ultracentrifugation. **(B)** MKN74-derived exosomes were validated by transmission electron microscopy (TEM). Scale bar: 200 nm. **(C)** Size distribution of exosomes was analyzed by Nanosight. **(D)** CD9 levels on the surface of exosomes were detected by flow cytometry analysis. Data represent mean ± SD, and Student's *t*-test was used to evaluate the statistical significance (***P < 0.001). **(E)** Western blot analysis of exosomes biomarker CD9 and CD63, and exosomal PD-L1 amounts. Equal amounts of proteins obtained from exosomes were immunoblotted with anti-CD9, anti-CD63, and anti-PD-L1 antibodies.

Accumulating evidence indicates that various cancer cells secrete exosomal PD-L1, including breast cancer, melanoma, prostate cancer, and glioblastoma (18–20, 29), which might play a crucial role in tumor progression. Our previous study showed that human gastric cancer cells also produce exosomal PD-L1 (21). However, whether 5-FU alters exosomal PD-L1 expression is unclear. To address this, we incubated MKN74 cells with 5-FU (0, 0.1, 1.0 μg/ml) for 48 and 72 h, respectively, and isolated MKN74-cell-derived exosomes from the supernatant by ultracentrifugation (Figure 2A). Exosomes confirmed by transmission electron microscopy (TEM) were limited by bilayer membranes with a characteristic ovoid or round shape (Figure 2B). NanoSight measurement showed that extracellular vesicles were ~107.4 nm in diameter (Figure 2C). A large proportion of exosomes were positive for the biomarker CD9, as detected by flow cytometry analysis (Figure 2D). Next, PD-L1 amounts in exosomes were examined in samples with similar levels of the exosome biomarkers CD9 and CD63. Exosomal PD-L1 levels were increased significantly after 5-FU treatment, with maximum value after treatment at 1.0 μg/ml 5-FU for 48 h and 0.1 μg/ml for 72 h (Figure 2E), indicating that 5-FU caused gastric cancer cells to produce exosomal PD-L1.

### Effects of Fluoropyrimidine on Circulating Exosomal PD-L1 and the Immune Status in Gastric Cancer Patients

**Figure 3:**
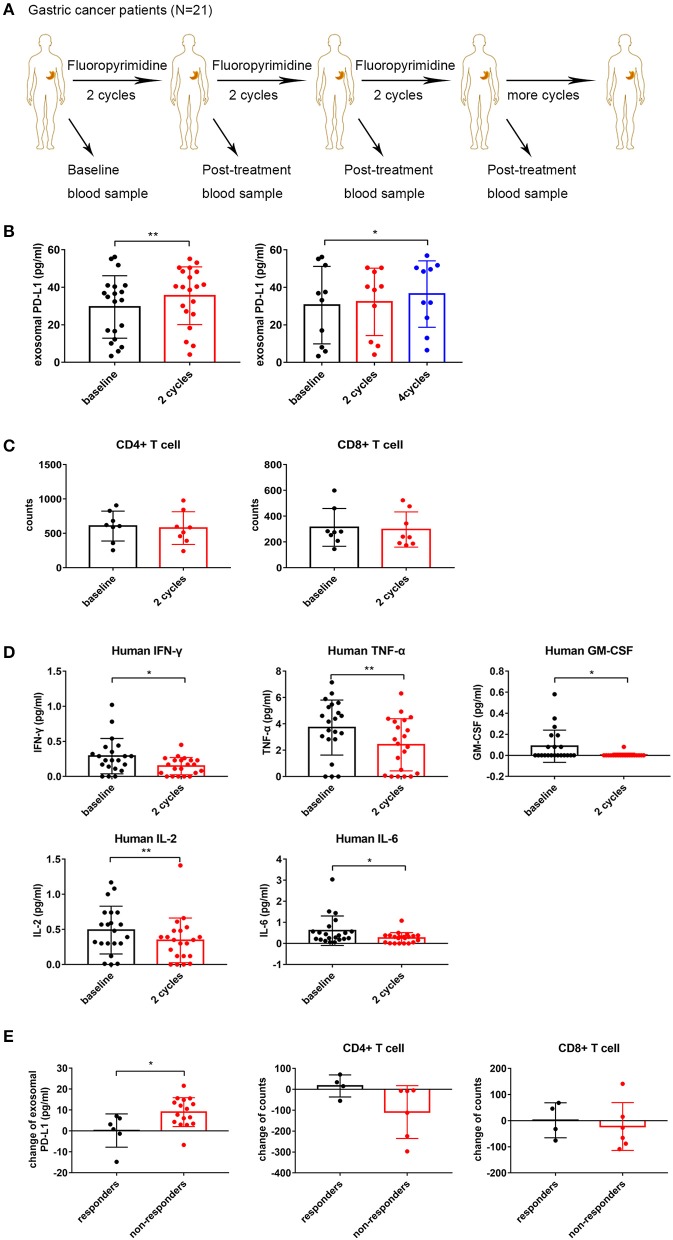
Fluoropyrimidine increases circulating exosomal programmed death-ligand 1 (PD-L1) in gastric cancer patients. **(A)** Schematic illustration of blood samples collection at baseline and after two, four, and six repeated cycles of fluoropyrimidine chemotherapy treatment from stage III–IV gastric cancer patients (*N* = 21). **(B)** Plot of circulating exosomal PD-L1 levels in gastric cancer patients at baseline and after two cycles (*N* = 21, one outlier was deleted) and four cycles (*N* = 10) of fluoropyrimidine chemotherapy treatment. **(C)** Left: Plot of the absolute counts of CD4^+^ T cells. Right: Plot of the absolute counts of CD8^+^ T cells (*N* = 8). **(D)** The levels of interferon-γ (IFN-γ), tumor necrosis factor-α (TNF-α), interleukin (IL)-2, IL-6, and granulocyte-macrophage colony stimulating factor (GM-CSF) in plasma were analyzed by multiplex bead assay at baseline and after two cycles of fluoropyrimidine chemotherapy. **(E)** Plot of changes of circulating exosomal PD-L1 levels and the absolute counts of T cells in responders and non-responders. The two-tailed paired *t*-test was used in statistical analysis where appropriate to evaluate the statistical significance (*P < 0.05, **P < 0.01).

Previous studies assessing the effects of 5-FU on PD-L1 expression are limited to mouse models. The effects of 5-FU on exosomal PD-L1 have been neglected, while much attention has been paid to the membrane form. It is essential to evaluate the change in circulating exosomal PD-L1 levels following multiple cycles of 5-FU treatment in advanced gastric cancer patients. We obtained matched blood samples at baseline and after different cycles of chemotherapy from 21 stage III–IV gastric cancer patients, who only received fluoropyrimidine standardized chemotherapy (including 5-FU, Capecitabine or S-1) (Figure 3A). Compared with baseline levels, circulating exosomal PD-L1 was upregulated after two (*N* = 21, *P* = 0.009) and four (*N* = 10, *P* = 0.047) cycles of fluoropyrimidine treatment (Figure 3B). There were only three patients with blood samples after six cycles of chemotherapy, who showed upregulated circulating exosomal PD-L1 (*N* = 3, *P* = 0.023) (Figure S1A).

To further investigate the immune status after different cycles of chemotherapy, we analyzed the absolute counts of T cell subgroups and the levels of cytokines in gastric cancer patients. The absolute counts of CD4^+^ and CD8^+^ T cells showed a reduction trend but without statistical significance (*N* = 8; *P* = 0.4023 and *P* = 0.4893, respectively) (Figure 3C). As shown in Figure 3D, IFN-γ, TNF-α, IL-2, IL-6, and GM-CSF amounts were decreased significantly after two cycles of treatment (*P* = 0.014, *P* = 0.004, *P* = 0.009, *P* = 0.031, and *P* = 0.014, respectively), but all were recovered at different degrees after four and six cycles of treatment (Figure S1B). The changes in exosomal PD-L1 was not associated with any changes of all eight immune cytokines in the clinical blood samples after two and four cycles of treatment (*P* > 0.05) (Tables S1, S2). The sample size of patients with six cycle treatments (*N* = 3) was too small to conduct any correlation analysis. These results suggested that repeated cycles of fluoropyrimidine treatment not only increased circulating exosomal PD-L1 amounts but also impaired the immune status and functions by inhibiting cytotoxic cytokines.

Next, we determined whether the change of circulating exosomal PD-L1 levels was associated with the treatment efficacy of fluoropyrimidine. Objective response rate (ORR) is an important indicator for evaluating the effectiveness of antitumor drugs. Based on that, among 21 gastric cancer patients, those showing treatment efficacy as complete response (CR), or PR were considered responders. Meanwhile, patients whose treatment efficacy was SD or PD were considered non-responders. We observed that circulating exosomal PD-L1 amounts were elevated obviously in non-responders while only slightly increasing or even decreasing in responders (*P* = 0.018). Meanwhile, the absolute counts of CD4^+^ and CD8^+^ T cells were decreased more significantly in non-responders compared with responders, although no statistically significant differences were observed (*P* = 0.1025 and *P* = 0.6689, respectively) (Figure 3E). Additionally, the changes in cytokines showed no differences between responders and non-responders (Figure S1C).

### Exosomal PD-L1 Induces Jurkat T Cell Apoptosis and Suppresses T Cell Activation in PBMCs

**Figure 4:**
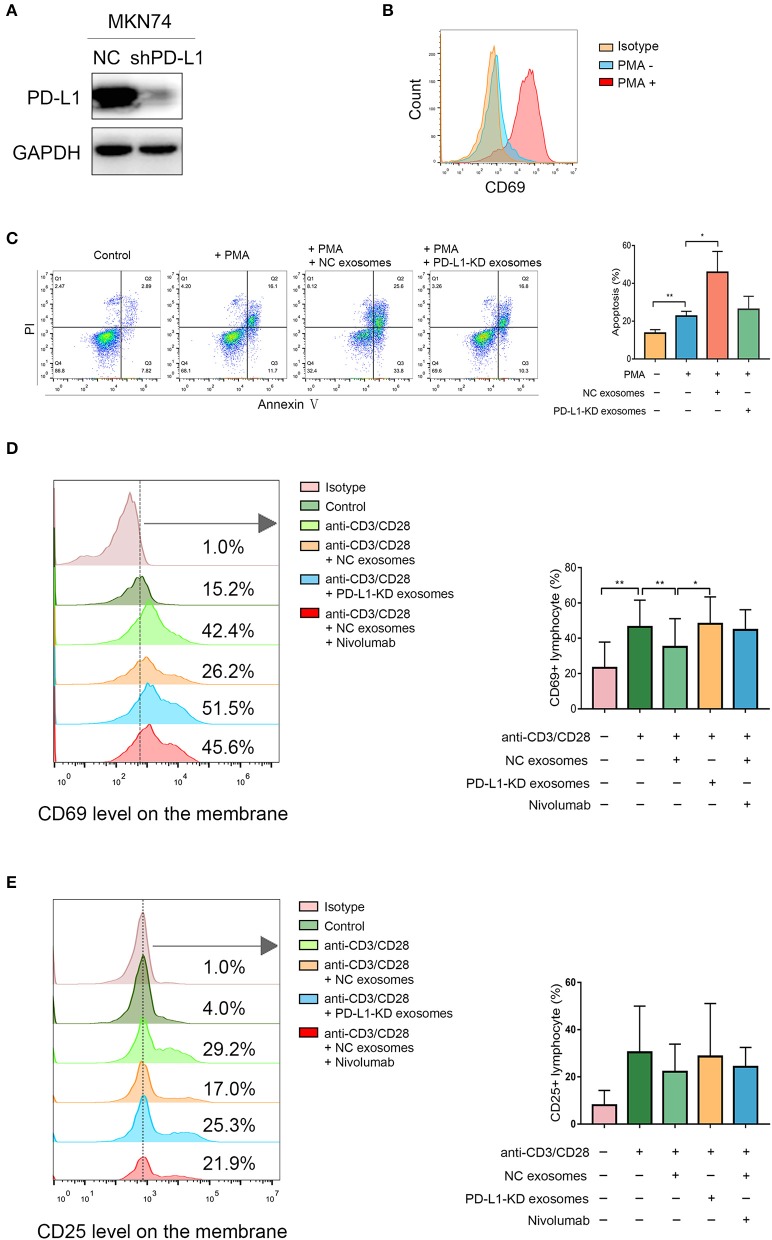
Exosomal programmed death-ligand 1 (PD-L1) induces Jurkat T cell apoptosis and suppresses T cell activation in peripheral blood mononuclear cells (PBMCs). **(A)** Western blot showed knockdown effect of PD-L1 in MKN74 cells. **(B)** Jurkat T cells were treated with 50 ng/ml phorbol 12-myristate 13-acetate (PMA) for 24 h, and activated Jurkat T cells were verified by high expression of CD69 on the surface using flow cytometry analysis. **(C)** Activated Jurkat T cells were coincubated with exosomes from PD-L1-NC and PD-L1-KD groups for 48 h, respectively. The apoptosis in activated Jurkat T cells was measured by flow cytometry analysis. **(D,E)** PBMCs were isolated from healthy human volunteers (*N* = 3). T cells in PBMCs were activated by human CD3/CD28 T cell activator for 6 h at 37°C and then coincubated with exosomes from PD-L1-NC and PD-L1-KD groups for 48 h. Activated T cells in PBMC exposure to NC exosomes in the presence or absence of anti-PD-L1 antibody nivolumab for 16 h. The activation markers CD69 and CD25 levels were analyzed by flow cytometry. Statistical analysis was performed by one-way ANOVA. (*P < 0.05, **P < 0.01). Data were representative of three independent experiments.

We aimed to assess whether exosomal PD-L1 derived from human gastric cancer has suppressive effects on T cell function, similar to PD-L1 found in the membrane of tumor cells. The expression of PD-L1 was obviously lower in PD-L1-KD MKN74 cells compared with that in PD-L1-NC MKN74 cells (Figure 4A). Jurkat T cells were activated by 50 ng/ml PMA, as verified by significant upregulation of CD69 on the cell surface (Figure 4B). As shown in Figure 4C, apoptosis in activated Jurkat T cells was enhanced significantly after exposure to exosomes presenting PD-L1 compared with PD-L1-KD exosomes. Given its ability to induce T cell apoptosis, whether exosomal PD-L1 inhibits T cell activation in PBMCs was assessed. After exposure to exosomes derived from the NC group, cells expressing CD69, an “early” activation marker, showed a more pronounced percentage decrease (Figure 4D). In addition, the fraction of cells positive for CD25, a “late” activation marker, also showed a decreasing trend, although statistical significance was not achieved (Figure 4E). To ensure that the above observations were specific to PD-L1, we next included the anti-PD1 antibody nivolumab (0.1 μg/ml) in the assays to block PD-1 signaling after T cell exposure to NC exosomes. As expected, CD69 and CD25 amounts on T cells were increased to levels comparable to those obtained after exposure to PD-L1-KD exosomes (Figures 4D,E). Taken together, these data showed that exosomal PD-L1 in human gastric cancer had a direct effect in inhibiting the functions of T cells, which could be attenuated by nivolumab.

### MiR-940/Cbl-b/STAT5A Are Involved in 5-FU-Induced Upregulation of PD-L1 in Gastric Cancer Cells

**Figure 5:**
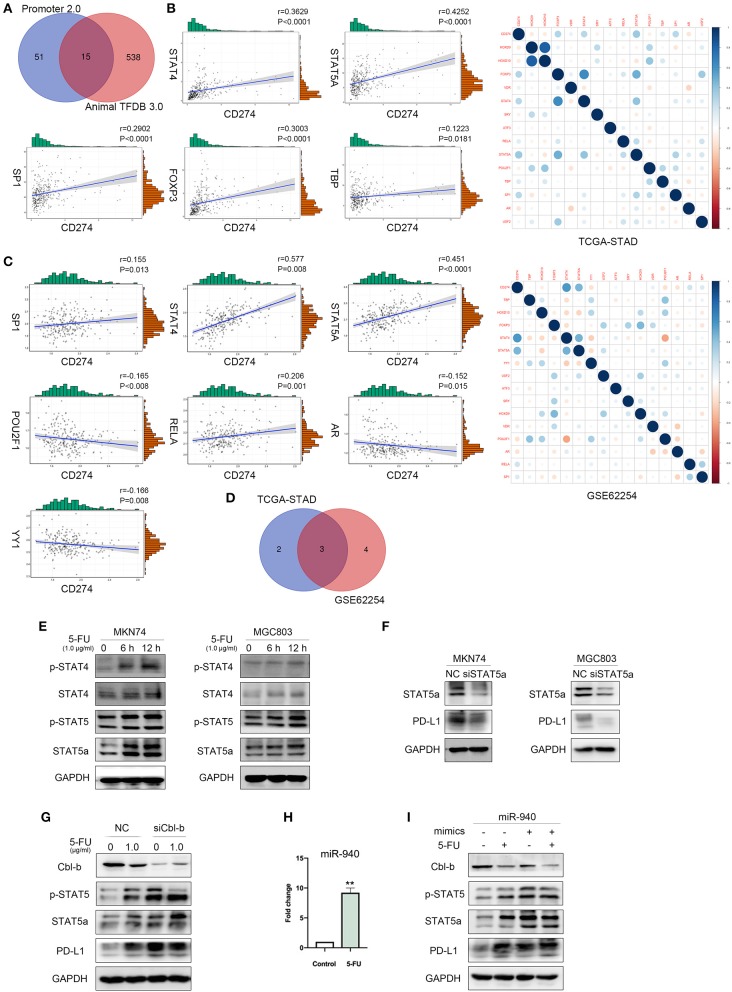
MiR-940/Cbl-b/signal transducer and activator of transcription 5A (STAT5A) are involved in 5-fluorouracil (5-FU)-induced upregulation of programmed death-ligand 1 (PD-L1) in gastric cancer cells. **(A)** Online database Promoter 2.0 Prediction Server and Animal TFDB 3.0 predicted transcriptional factors of PD-L1. **(B–D)** Correlation analysis of predicted transcriptional factors with PD-L1 using the gene expression profiles of TCGA-STAD database and GSE62254. **(E)** MKN74 and MGC803 cells were treated with 1.0 μg/ml 5-FU for 6 or 12 h; the phosphorylation of signal transducer and activator of transcription 4 (STAT4) and STAT5A were analyzed by Western blot. **(F)** After transient knockdown of STAT5A by using small interfering RNA (siRNA) for 72 h, STAT5A and PD-L1 levels were detected by Western blot analysis, and glyceraldehyde 3-phosphate dehydrogenase (GAPDH) was used as internal control. Data were representative of three independent experiments. **(G)** After transient knockdown of Cbl-b by using siRNA for 72 h and treated MGC803 with 1.0 μg/ml 5-FU for 72 h, simultaneously, Cbl-b, p-STAT5, STAT5A, and PD-L1 levels were detected by Western blot analysis. **(H)** The expression of miR-940 in MGC803 treated with or without 1.0 μg/ml 5-FU for 72 h was analyzed. **P < 0.01. **(I)** MGC803 was cotransfected with 40 nmol miR-940 mimics and treated with 1.0 μg/ml 5-FU for 72 h. Western blot analysis the expression of Cbl-b, p-STAT5, STAT5A, and PD-L1 in MGC803.

Through the online database UCSC Genome Browser, we predicted sequences in the promoter region of PD-L1. Promoter 2.0 Prediction Server and Animal TFDB 3.0 were utilized to predict transcription factors according to the promoter sequences and 15 transcriptional factors were mutually identified to possibly participate in PD-L1 regulation (Figure 5A). We performed correlation analysis between these transcription factors and PD-L1. Results with TCGA-STAD dataset showed that five transcriptional factors were associated with PD-L1, including FOXP3 (*r* = 0.3003, *P* < 0.0001), STAT4 (*r* = 0.3629, *P* < 0.0001), STAT5A (*r* = 0.4252, *P* < 0.0001), TBP (*r* = 0.1223, *P* = 0.0181), and SP1 (*r* = 0.2902, *P* < 0.0001) (Figure 5B). Results with GSE62254 dataset in gastric cancer in the NCBI-GEO database showed that seven predicted transcriptional factors were associated with PD-L1, including STAT4 (*r* = 0.577, *P* < 0.0001), STAT5A (*r* = 0.451, *P* < 0.0001), YY1 (*r* = −0.166, *P* = 0.008), POU2F1 (*r* = −0.165, *P* = 0.008), AR (*r* = −0.152, *P* = 0.015), RELA (*r* = 0.206, *P* = 0.001), SP1 (*r* = 0.155, *P* = 0.013) (Figure 5C). We made an intersection between the two cohorts and identified three transcriptional factors of PD-L1, including SP1, STAT4, and STAT5A (Figure 5D). Based on their high correlation coefficients (*r* > 0.3), we selected STAT4 and STAT5A for further experiments. Western blot was performed to measure the expression of STAT4 and STAT5A and their phosphorylation levels in MKN74 and MGC803 cells after treatment with 1.0 μg/ml 5-FU for 6 and 12 h. As shown in Figure 5E, 5-FU significantly increased STAT4, STAT5A, and their phosphorylation levels after treatment for 12 h. Since the expression of STAT4 in gastric cancer cells is low or undetectable, we evaluated the correlation of STAT5A and PD-L1 in the further investigation. After STAT5A knockdown by siRNA, PD-L1 expression levels in MKN74 and MGC803 were downregulated (Figure 5F). Our previous study revealed that the miR-940/Cbl-b/STAT5A axis might regulate PD-L1 in gastric cancer cells (30). Given that Cbl-b could directly interact with STAT5A and increase the ubiquitination of STAT5A (30), we further examined whether the Cbl-b is involved in 5-FU-induced upregulation of STAT5A and PD-L1 in gastric cancer cells. Due to the higher expression of Cbl-b in MGC803, we silenced Cbl-b with siRNA followed by 5-FU treatment. Western blot detected the expressions of Cbl-b, STAT5A, and PD-L1. The results showed that Cbl-b expression was decreased after 5-FU treatment. Meanwhile, silencing of Cbl-b increased 5-FU-induced upregulation of p-STAT5 and PD-L1 (Figure 5G), indicating that 5-FU activated STAT5A and increased PD-L1 expression through downregulation of Cbl-b. Given that miR-940 was found in the 3′-untranslated region (UTR) of Cbl-b transcript (30), we detected the expression of miR-940 in MGC803. Quantitative RT-PCR analysis showed that the relative level of miR-940 was significantly increased in MGC803 cells following 5-FU treatment (*P* < 0.01; Figure 5H). Next, in order to investigate the effect of miR-940 on 5-FU-induced upregulation of PD-L1 expression, MGC803 cells were transfected with mimics of miR-940 or negative control followed by 5-FU treatment. The results showed that transfected miR-940 mimics further decreased 5-FU-induced Cbl-b expression, whereas p-STAT5 and PD-L1 were increased (Figure 5I). Taken together, these results indicated that 5-FU-induced upregulation of PD-L1 is mediated by miR-940/Cbl-b/STAT5A in gastric cancer cells.

## Discussion

Chemotherapy is the conventional treatment for advanced cancer but shows a limited survival advantage. Immunotherapy, which is characterized by immune checkpoint blockade (ICB), has changed the standard of care for a subgroup of patients and improved survival compared with previous criteria in several cancer types, even though limitations remain (31, 32). The efficacy of chemotherapy combined with ICB has been evaluated by several preclinical studies and clinical trials in the recent years. Combination therapies successfully provided survival benefit for patients in some cancer types, such as non-small-cell lung carcinoma (NSCLC), while they failed in other cancer types, such as gastric cancer (33–35). The KEYNOTE-062 clinical study found that pembrolizumab plus chemotherapy as the first-line therapy did not show superior overall survival (OS) and progression-free survival (PFS) in advanced gastric cancer (36). However, the molecular mechanism underlying this combined treatment requires further elucidation. In the present study, our results indicated that 5-FU treatment could exert immunosuppressive effects through upregulation of PD-L1 expression in gastric cancer. This might be a reason that patients have not benefited from combination therapy.

Increasing studies have focused on the role of exosomal PD-L1 in the recent years (37). Exosomes could transfer functional PD-L1 locally and distantly to suppress T cell activation and proliferation (18, 19). Previous study indicates that deletion of exosomal PD-L1 results in significantly decrease percentage of Tim3^+^ cells and increased Granzyme B T cells in mouse prostate cancer models (20). In addition, exosomal PD-L1 inhibits the proliferation of CD8^+^ T cells, as reflected by decreased expression levels of Ki-67 and Granzyme B, which promotes the progression of melanoma both *in vivo* and *in vitro* (19). Consistent with the suppressive effects of exosomal PD-L1 on T cell function, our study suggested that MKN74-cell-derived exosomal PD-L1 induced apoptosis in Jurkat T cells and inhibited T cell activation in PBMCs, which could be attenuated by nivolumab. Few studies demonstrated that there is a significant correlation between high level of circulating exosomal PD-L1 with clinicopathological characters such as advanced tumor stage, disease progression, and lymph node involvement (20, 38). Our previous study has demonstrated that high exosomal PD-L1 was an independent prognostic factor, which was significantly associated with advanced tumor stage and poor overall survival (21). The study published in *Nature* in 2018 indicated that circulating exosome PD-L1 increased in clinical responders undergoing pembrolizumab (19). However, no studies reported the effects of chemotherapy on circulating exosomal PD-L1 in clinical responders and non-responders. Importantly, our results indicated that circulating exosomal PD-L1 in gastric cancer patients increased significantly after two, four, and six cycles of fluoropyrimidine treatment, and the increase in circulating exosomal PD-L1 was more pronounced in non-responders than in responders. These findings suggest that upregulation of circulating exosomal PD-L1 might be a mechanism by which 5-FU induced immunosuppression in gastric cancer.

Cytotoxic cytokines play an important role in promoting antitumor immunity. IFN-γ is a critical cytokine secreted by activated T cells and plays crucial roles in promoting antitumor immune responses (39). TNF-α produced by various cell types, including lymphocytes and macrophages, is highly involved in the regulation of immune responses (40). IL-2 is termed as a key positive regulator of CD4^+^ T cell differentiation and function and can regulate the effector and memory responses of CD8^+^ T cells (41). GM-CSF was reported to inhibit bladder cancer growth by reducing lymphangiogenesis and recruitment of M2 macrophages (42). Our results showed that several cytokines such as IFN-γ, TNF-α, IL-2, IL-6, and GM-CSF were significantly reduced in the plasma of gastric cancer patients following repeated cycles of fluoropyrimidine treatment, especially after two cycles, suggesting that the functions of T cells and immune responses were impaired by fluoropyrimidine. Previous study has reported that exosomal PD-L1 derived from tumor cells inhibited the proliferation, cytokine production, and cytotoxicity of CD8^+^ T cells (19). However, results of our study showed that exosomal PD-L1 was not associated with the changes in immune cytokines after two or four cycles of fluoropyrimidine treatment. Considering that circulating PD-L1^+^ exosomes were secreted not only by tumor cells but also by antigen-presenting cells (APCs), it was unclear whether PD-L1^+^ exosomes produced by APC affect the immune response like exosomes derived from tumor cells (38). In addition to the aforementioned findings, our data showed that the absolute counts of CD4^+^ and CD8^+^ T cells decreased following fluoropyrimidine treatment, although the change was not statistically significant. Previous study has reported similar results as the CD4^+^ and CD8^+^ T cell amounts could recover quickly after chemotherapy, with mean CD8^+^ T cell amounts returning to baseline more quickly than CD4^+^ T cell levels (43). Consistent with findings in CT26 tumor-bearing mouse model (44), this study with clinical blood samples suggested that inhibition of key cytokines production might be a second mechanism by which 5-FU induced immunosuppression in gastric cancer.

Various regulatory mechanisms of PD-L1 expression have been studied, mainly including (1) gene alteration at the PD-L1 locus, (2) control of PD-L1 gene expression through inflammatory signaling, (3) aberrant oncogenic signaling influences PD-L1 expression, (4) miRNA-mediated PD-L1 mRNA regulation, and (5) PD-L1 regulation at protein level (45–50). However, few studies have reported the mechanism of PD-L1 expression by 5-FU induction. It was reported that Folfox (5-FU plus oxaliplatin) increases PD-L1 levels by IFN-γ secreted by Folfox-induced CD8^+^ T cells (51). In the present study, our results suggest that 5-FU-induced upregulation of PD-L1 might be mediated by miR-940/Cbl-b/STAT5A axis in gastric cancer cells.

In summary, we demonstrated that 5-FU upregulated gastric cancer cell-derived exosomal PD-L1 in a dose- and time-dependent manner. Fluorouracil chemotherapy might exert immunosuppressive effects by upregulating exosomal PD-L1, accompanied by a decrease in cytokines in gastric cancer patients. Exosomal PD-L1 derived from gastric cancer cells induced apoptosis of Jurkat T cells and inhibited T cells activation in PBMCs. Additionally, our data suggested that 5-FU upregulated PD-L1 expression through miR-940/Cbl-b/STAT5A axis in gastric cancer cells. These results provide new insights into immunosuppressive effects of 5-FU. However, this study was limited due to the lack of a mouse model to validate the relationship between exosome PD-L1 and tumor immunity *in vivo* and whether inhibiting exosomal PD-L1 can improve immune activation targeting GC.

## Data Availability Statement

Our experimental data are unpublished data in the past and do not include data from other authors or other organizations. Part of the data come from the public free-charged database. The source of database has been added to the article.

## Ethics Statement

The study was reviewed and approved by the Human Ethics Review Committee of the First Hospital of China Medical University, and all researches were conducted in accordance with ethical principles. All patients agree to participate in our study.

## Author Contributions

MZ and YF performed the experiments, analyzed the data, and wrote the manuscript. XC designed experiments and revised the manuscript. XQ and YL supervised the studies, obtained funding, and also revised the paper. KH, CZ, TW, CL, SW, and YC performed experiments. All authors read and approved the final manuscript.

## Conflict of Interest

The authors declare that the research was conducted in the absence of any commercial or financial relationships that could be construed as a potential conflict of interest.

## Supplementary Material

Figure S1 **(A)** Plot of circulating exosomal PD-L1 levels in gastric cancer patients at baseline and after six cycles of fluoropyrimidine chemotherapy treatment (*N* = 3). **(B)** The levels of IFN-γ, TNF-α, IL-2, IL-6, and GM-CSF in plasma were analyzed by multiplex bead assay at baseline and after four and six cycles of fluoropyrimidine chemotherapy. **(C)** Plot of changes of IFN-γ, TNF-α, IL-2, IL-6, and GM-CSF levels in plasma in responders and non-responders groups. The two-tailed paired *t*-test was used in statistical analysis where appropriate to evaluate the statistical significance (^*^*P* < 0.05, ^**^*P* < 0.01).

Tables S1 and S2 Correlation between the changes of exosomal PD-L1 and eight immune cytokines in the clinical blood samples after two and four cycles of treatment.

## Abbreviations

5-FU: 5-fluorouracil
GC: gastric cancer
TS: thymidylate synthase
ICD: immunogenic cell death
PD-L1: programmed death-ligand 1
PD-1: programmed death-1
EVs: extracellular vesicles
IL-2: interleukin-2
IL-4: interleukin-4
IL-6: interleukin-6
IL-8: interleukin-8
IL-10: interleukin-10
GM-CSF: granulocyte-macrophage colony stimulating factor
TNF-α: tumor necrosis factor-α
IFN-γ: interferon-γ
ORR: objective response rate
PR: partial response
SD: stable disease
PD: progressive disease
PBMCs: peripheral blood mononuclear cells
PMA: phorbol 12-myristate 13-acetate
TCGA: The Cancer Genome Atlas
STAT4: signal transducer and activator of transcription 4
STAT5A: signal transducer and activator of transcription 5A
siRNA: small interfering RNA
shRNA: short hairpin RNA
NC: negative control.
