# Supplementary figures and images for: 5-FU-Induced Upregulation of Exosomal PD-L1 Causes Immunosuppression in Advanced Gastric Cancer Patients
Source: Front Oncol. 2020 Apr 22;10:492. doi: 10.3389/fonc.2020.00492 (PMC7188923; doi:10.3389/fonc.2020.00492)

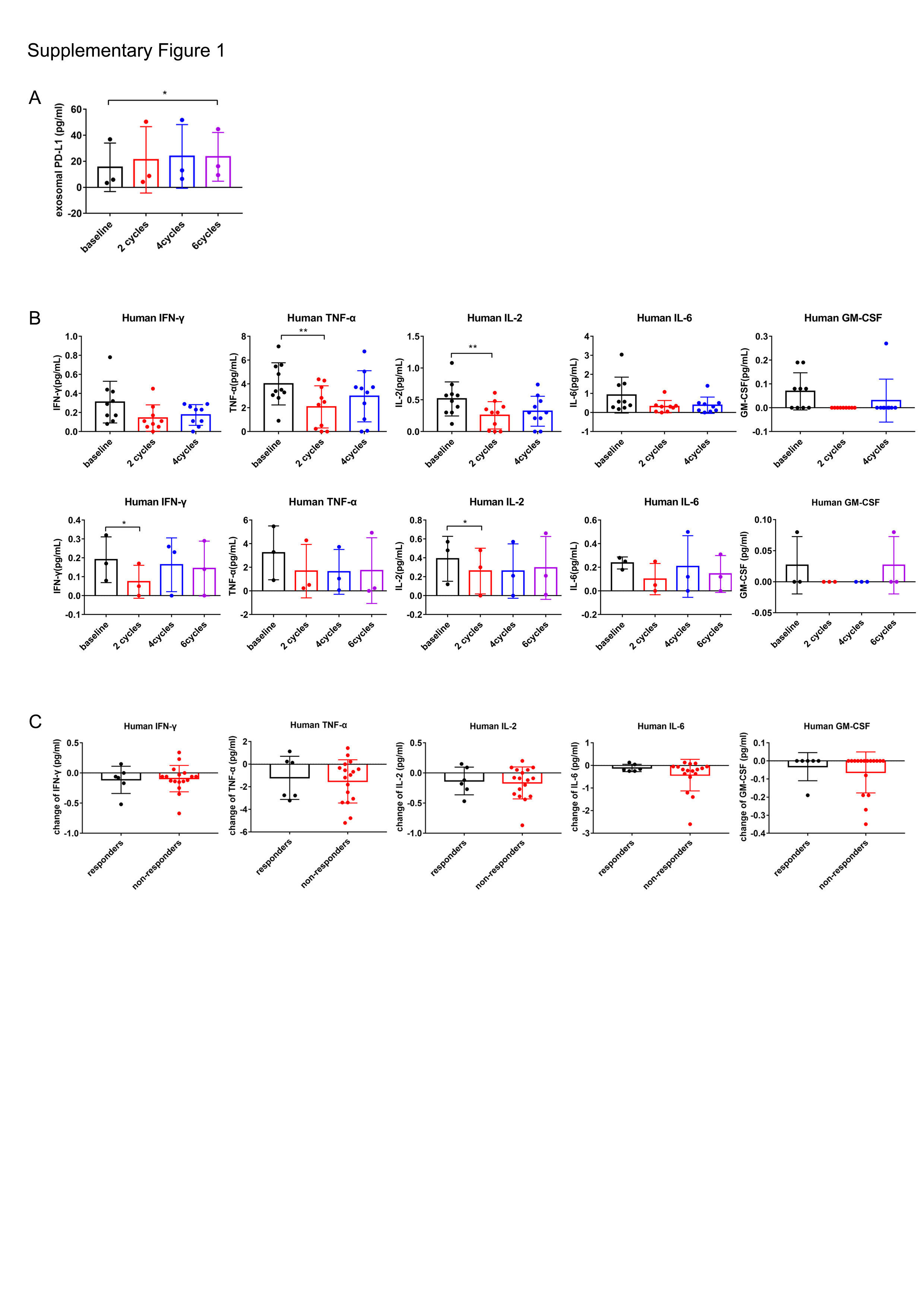

Supplement: Figure S1 — (A) Plot of circulating exosomal PD-L1 levels in gastric cancer patients at baseline and after six cycles of fluoropyrimidine chemotherapy treatment (N = 3). (B) The levels of IFN-γ, TNF-α, IL-2, IL-6, and GM-CSF in plasma were analyzed by multiplex bead assay at baseline and after four and six cycles of fluoropyrimidine chemotherapy. (C) Plot of changes of IFN-γ, TNF-α, IL-2, IL-6, and GM-CSF levels in plasma in responders and non-responders groups. The two-tailed paired t-test was used in statistical analysis where appropriate to evaluate the statistical significance (*P < 0.05, **P < 0.01). [file Image_1.TIF]
