# Supplementary material for: 5-FU-Induced Upregulation of Exosomal PD-L1 Causes Immunosuppression in Advanced Gastric Cancer Patients
Source: Front Oncol. 2020 Apr 22;10:492. doi: 10.3389/fonc.2020.00492 (PMC7188923; doi:10.3389/fonc.2020.00492)
Supplement: Tables S1 and S2 — Correlation between the changes of exosomal PD-L1 and eight immune cytokines in the clinical blood samples after two and four cycles of treatment. [file Table_1.DOCX]

Table S1

The correlation between the changes of exosomal PD-L1 and cytokines at 2 cycles (N=21)

| Name | Spearman correlation coefficient | P-value |
| --- | --- | --- |
| ΔIL-2 | 0.439 | 0.053 |
| ΔIL-4 | 0.323 | 0.165 |
| ΔIL-6 | 0.266 | 0.256 |
| ΔIL-8 | 0.189 | 0.424 |
| ΔIL-10 | -0.097 | 0.685 |
| ΔGM-CSF | 0.253 | 0.283 |
| ΔIFN-γ | 0.389 | 0.090 |
| ΔTNF-α | 0.318 | 0.171 |

Table S2

The correlation between the changes of exosomal PD-L1 and cytokines at 4 cycles (N=10)

| Name | Spearman correlation coefficient | P-value |
| --- | --- | --- |
| ΔIL-2 | -0.061 | 0.868 |
| ΔIL-4 | 0.410 | 0.240 |
| ΔIL-6 | -0.213 | 0.555 |
| ΔIL-8 | 0.358 | 0.310 |
| ΔIL-10 | 0.522 | 0.122 |
| ΔGM-CSF | -0.013 | 0.972 |
| ΔIFN-γ | 0.206 | 0.567 |
| ΔTNF-α | 0.030 | 0.934 |
